# Bilateral Vocal Fold Paralysis in Myasthenia Gravis: A Case Report and Literature Review

**DOI:** 10.3389/fneur.2020.581060

**Published:** 2020-10-15

**Authors:** Christopher Nelke, Bendix Labeit, Sven G. Meuth, Tobias Warnecke, Rainer Dziewas, Tobias Ruck

**Affiliations:** Clinic of Neurology With Institute of Translational Neurology, University Hospital Münster, Münster, Germany

**Keywords:** bilateral vocal fold paralysis, myasthenia gravis, myasthenic crisis, case report, literature review

## Abstract

To prevent severe and potentially life-threatening consequences of bilateral vocal fold paralysis (BVFP), the identification and management of reversible causes is pivotal. Myasthenia gravis (MG) presenting with BVFP is rarely reported and remains incompletely understood. Although symptom control is achievable for most MG patients with sufficient therapy, atypical clinical presentation such as BVFP might preclude diagnosis and thus effective treatment. Here, we present a case of BVFP as leading manifestation of MG successfully treated with plasmapheresis. Moreover, we performed a literature review of the few existing cases reported between 1980 and 2020 indicating that elderly patients are particularly at risk for MG presenting with severe BVFP and that edrophonium testing with fiber optic endoscopic evaluation of swallowing (FEES) might be valuable for establishing the diagnosis. We conclude that clinicians should consider MG as possible and reversible cause for BVFP.

## Introduction

Bilateral vocal fold paralysis (BVFP) is a life-threatening condition. Management and diagnosis of BVFP often constitutes a clinical challenge impeded by a broad range of potential etiologies (1). However, identification of reversible causes is critical for patient management and prevention of irreversible damages as BVFP often requires urgent airway management such as intubation or tracheotomy (2). For adults, the most common causes for BVFP are tumors, surgery, and idiopathic (meaning no cause has been identified) vocal cord paralysis (3). However, underlying neurological conditions, such as cranial neuropathies or ischemic stroke, are frequently observed in patients presenting with idiopathic vocal cord paralysis (4). Of note, myasthenia gravis (MG) as a possible cause for BVFP is only rarely reported and a systematic analysis of the topic is lacking. Here, we report a patient presenting with BVFP as leading clinical manifestation of MG resulting in severe respiratory dysfunction. Intensive diagnostic work-up finally allowed for the diagnosis of MG and FEES edrophonium testing was key to the diagnosis. We further analyzed the available cases of MG patients presenting with BVFP by performing a literature review to understand and characterize this specific subgroup.

## Case Report

An 80 year old Caucasian woman was admitted due to rapidly progressive dyspnea and dysarthria to a tertiary care hospital. The patient reported slight dyspnea, difficulty of swallowing, and hoarseness of voice 3 days before admission. The patient recalled no precipitating factors, including infections. The prior medical history was unremarkable with no known preceding illness. On admission, the patient presented with severe dyspnea already in resting state and inspiratory stridor. Articulation of speech was severely restricted due to dysarthria and dysphonia with the latter as leading component of speech impairment. Clinical examination revealed mild, bilateral ptosis aggravated by sustained upgaze and fatigable diplopia in sustained lateral gaze. Counting aloud revealed fatigable paresis of the tongue and aggravated dysphonia. Other muscles with cranial innervation were not affected retaining full motor function. No fatigable weakness of limb muscles was detected with sustained abduction of the arms or elevation of the legs (>120 s) possible although examination was impeded due to dyspnea. Similarly, individual testing of limb muscles revealed no paresis or fatigable weakness. Reflexes (biceps, triceps, patellar, and tendocalcaneus) and sensitivity to touch were intact. Cranial CT imaging was unremarkable. Due to progressive symptoms, the patient was transferred 3 days later to our neurological intensive care unit. Here, clinical examination confirmed previous findings; however respiratory dysfunction proceeded with progressive inspiratory stridor. Weakness of the respiratory or diaphragmatic muscles was not observed during clinical examination. Fiber optic endoscopic evaluation of swallowing (FEES) ascertained severe neurogenic dysphagia with pooling of saliva in the valleculae and the piriform sinus, bilateral abductor paralysis of the vocal cords (see Supplementary Video 1), paralysis of the pharyngeal constrictors and a missing swallow-reflex. When testing small liquid boli, pre-and intradeglutitive aspiration was noted [ranked as 7 according to the penetration aspiration scale of (5)]. These findings were clearly ameliorated upon intravenous application of 10 mg edrophonium applicated while monitoring vital sign (see Supplementary Video 1) (6). At this point, airway obstruction remained the leading clinical symptom with only minor affection of the respiratory muscle strength. Arterial blood gas analysis revealed severe CO_2_-retention compatible with limited expiration. Progressive respiratory insufficiency due to BVFP made invasive airway management necessary and endotracheal intubation was performed on the same day. Subsequently, a dilatational tracheotomy was performed. The further diagnostic work-up showed elevated acetylcholine receptor (AChR) antibodies (60 nmol/L). A chest CT, although lacking iodine contrast, was suggestive for the presence of a thymoma. Given the high perioperative risk and the sufficient treatment response achieved by PLEX, follow up diagnostics and thymectomy were planned after stabilization of the patient. In order to substantiate the diagnosis, repetitive nerve stimulation of the right nasalis muscle was performed revealing a decremental response. Therefore, the diagnosis of myasthenia gravis was established and therapy with PLEX and corticosteroids were initiated resulting in a marked improvement of respiratory status and dysphagia (see Supplementary Video 1). Repeated PLEX cycles resulted in clinical stabilization ultimately allowing for decannulation and control of the myasthenic syndrome. Concurrently, arterial blood gas analysis revealed sufficient respiration with normalized CO_2_-values. The patient was supported by mechanical ventilation for a total of 17 days. Hospitalization was complicated by symptomatic atrial fibrillation detected during monitoring 1 week after decannulation with no observed relation to central catheter placement or removal, which was necessary for the application of PLEX. For control of tachycardia, high dose β-blocker (bisoprolol) were applied achieving frequency control. Two weeks after decannulation the patient suffered ventricular fibrillation while monitored. Imminent resuscitation was carried out for 30 min but remained futile and the patient unfortunately died 49 days after initial admission.

## Literature Review

We performed a literature search for the terms “myasthenia gravis,” “myasthenic crisis,” and “bilateral vocal cord/fold paresis/paralysis/palsy” including results from January 1980 to January 2020 (Table 1). We identified 11 cases of BVFP, including our own, which were attributed to MG. In these cases, the male to female ratio was 1:1.2, the median age of BVFP was 68 years. 45.5% of patients were anti-AChR-Ab positive, 18.1% were anti-Musk-Ab positive. 72.3% of patients presented with BVFP as first manifestation of MG. Nine patients responded positive to edrophonium testing, while two patients were not tested. Treatment regimens were heterogeneous with most patients receiving either cortisone and/or pyridostigmine. Only 2 patients (18.2%) received PLEX as rescue therapy. Seven patients (63.6%) experiencing BVFP due to MG required invasive airway management such as mechanical ventilation or tracheotomy. Median age for these patients was 76 years, while median age for patients with good clinical outcomes was 35 years. Only one patient requiring intensive care received PLEX.

**Table 1:** Overview of reported cases of MG presenting with BVFP.

**Age at onset of BVFP (age at diagnosis)**	**Gender**	**Antibody-status**	**Treatment**	**Outcome**	**References, year**	**Edrophonium testing**
46	Male	Anti-AChR-Ab	Pyridostigmine	Good	(7) 1992	Positive
82 (68)	Female	Seronegative	Cortisone	Intubated, mechanical ventilation	(8) 2002	Positive (by FEES)
61	Male	Seronegative	Cortisone + Pyridostigmine	Tracheotomy, died due to respiratory failure	(9) 1999	Positive
76	Male	Anti-AChR-Ab	Cortisone	Tracheotomy	(10) 2007	Positive (by FEES)
68	Male	Seronegative	Pyridostigmine	Intubated, mechanical ventilation	(11) 2011	Positive
61 (56)	Male	Anti-Musk-Ab	Cortisone	Tracheostomy	(12) 2007	Positive (by FEES)
72	Female	Anti-AChR-Ab	Cortisone, PLEX, Pyridostigmine	Good	(13) 1996	Positive (by FEES)
16	Female	Seronegative	Pyridostigmine	Good	(14) 1999	Not performed
24 (22)	Female	Anti-Musk-Ab	Pyridostigmine	Good	(15) 2008	Not performed
78	Female	Anti-AChR-Ab	Cortisone	Tracheostomy	(16) 2014	Positive (by FEES)
80	Female	Anti-AChR-Ab	PLEX, Cortisone, Pyridostigmine	Tracheotomy, died due to cardiac arrest	(case presented here) 2020	Positive (by FEES)

*anti-AChR-ab, anti–acetylcholine receptor antibody; FEES, fiber optic endoscopic evaluation of swallowing; PLEX, plasmapheresis; IVIG, intravenous immunoglobulin*.

## Discussion

Our patient presented with airway obstruction as leading symptom and only mild ptosis suggested an underlying neurological disease. Considering differential diagnosis, brain stem stroke might be considered due to the relatively sudden onset of dysarthria. A native CT revealed no intracranial pathologies including ischemia; however, it should be noted that a cranial MRI would have provided higher sensitivity and constitutes the preferred diagnostic for the detection of brain stem stroke. Moreover, polyneuropathies, such as variants of Guillain-Barre syndrome (GBS) or diphtheritic polyneuropathy, might be considered given the predominant affection of cranially innervated muscles and later cardiac involvement. The absence of areflexia and ataxia as well as the lack of characteristic electrophysiological findings suggestive for nerve affection render these diagnoses less likely. Moreover, preceding, or in the case of diphtheritic polyneuropathy apparent infections often seen in these pathologies were absent. Considering all clinical information, particularly diagnostic and serological findings, MG remained the priority diagnosis. In the intensive diagnostic work-up, FEES edrophonium testing was particularly helpful to guide the clinical management and diagnostic findings including anti-AChR-Ab, a decrement in repetitive nerve stimulation and detection of a thymoma confirmed MG diagnosis. Therefore, FEES edrophonium testing provided early evidence of the underlying disease and should be implemented in clinical standard operating procedures (SOP) for BVFP. While respiratory failure appears to be the main cause for mortality and the associated disease burden, our patient died due to comorbidity that was only indirectly attributable to the myasthenic crisis. Indeed, comorbidities might critically influence the outcome as older patients had worse outcome as compared to younger patients, which might be potentially attributed due to a higher frequency of comorbidities. While our case and Osei-Lah et al. reported death due to BVFP share severe respiratory insufficiency as leading symptom, a difference is that our patient received rescue therapy potentially ameliorating respiratory function underlining the importance for employing appropriate treatment strategies in these cases (9).

In the literature only few cases report on MG patients presenting with BVFP as leading clinical feature exist, while a systematic characterization of these patients is lacking. In these case reports, patients presenting with BVFP due to MG are often elderly, respond to edrophonium and display a detection rate for anti-Musk-Ab of 18.1%. However, the actual rate of anti-Musk-Ab positivity might be higher, given that many of the cases were described previous to the introduction of anti-Musk-Ab testing (17). MG patients with anti-Musk-Ab constitute a distinct clinical subgroup characterized by frequent ocular and/or bulbar symptoms and myasthenic crisis (18). Of note, the high rate of detection for anti-Musk-Ab in MG patients presenting with BVFP [18.1% in BVFP vs. 3–5% in most MG cohorts (19)] suggests that early and severe affection of the vocal folds might contribute to the incidence of myasthenic crisis requiring invasive airway management for anti-Musk-Ab MG patients (17, 20). Advanced age was associated with poor clinical outcome. As comorbidities were infrequently documented, it remains unclear whether this association is due to a different disease phenotype seen in late-onset MG and/or frequent comorbidities in elderly patients (21). Treatment regimens were heterogeneous and 7 out of 11 patients (63.6%) required intensive care of whom only one patient received PLEX suggesting that MG patients presenting with BVFP might remain insufficiently treated despite the establishment of diagnosis. Interestingly, despite the advanced age, PLEX was able to avert the need for invasive airway management in the 72 year old patient reported by Hanson et al. (13). Further studies are necessary for a conclusive statement regarding treatment options for MG patients presenting with BVFP. We suggest that patients presenting with BVFP and no clear etiology should receive neurological consultation, particularly as covert neurological conditions are frequent for these patients and potentially amendable (4). Besides clinical examination by a neurologist, edrophonium testing is highly valuable for the detection of MG as underlying cause of BVFP, in particular if combined with FEES (22, 23).

A strength of this study is the availability of video material illustrating the key clinical findings necessary to facilitate the diagnosis. Combined with the first characterization of this group of patients, we believe that the data presented here might improve management of BVFP. We recognize that the precocious death of the patient precludes the assessment of the follow-up outcome and that the missing patient perspective constitute a limitation of this study.

We conclude that MG should be considered as differential diagnosis for patients presenting with BVFP, given the high number of cases requiring intensive care despite the availability of effective treatment options. Neurological consultation combined with FEES edrophonium testing should be implemented in clinical SOPs for BVFP.

## Data Availability Statement

The original contributions generated in the study are included in the article/Supplementary Material, further inquiries can be directed to the corresponding author/s.

## Ethics Statement

Ethical review and approval was not required for the study on human participants in accordance with the local legislation and institutional requirements. The patients/participants provided their written informed consent to participate in this study. Written informed consent was obtained from the individual for the publication of any potentially identifiable images or data included in this article.

## Author Contributions

CN: design and conceptualized study and drafted manuscript. BL: generation of video material and revised the manuscript for intellectual content. SM and TW: revised the manuscript for intellectual content. RD and TR: design and conceptualized study and revised the manuscript for intellectual content. All authors contributed to the article and approved the submitted version.

## Conflict of Interest

The authors declare that the research was conducted in the absence of any commercial or financial relationships that could be construed as a potential conflict of interest. The handling editor declared a past co-authorship with authors TW and TR.

## Supplementary Material

Supplementary Video 1 Findings from fiber optic endoscopic evaluation of swallowing (FEES). (1) Severe neurogenic dysphagia with bilateral abductor paralysis (BVFP) of the vocal cords was recorded prior to application of edrophonium. (2) BVFP is ameliorated upon intravenous application of 10 mg edrophonium. Reduced movement of vocal cords during vocalization is still seen. (3) Findings after completing plasmapheresis (PLEX). Normal vocal cord movement during vocalization is seen. Status of dysphagia and ventilation is vastly improved.
